# Genome sequence and Carbohydrate Active Enzymes (CAZymes) repertoire of the thermophilic *Caldicoprobacter algeriensis *TH7C1^T^

**DOI:** 10.1186/s12934-022-01818-0

**Published:** 2022-05-21

**Authors:** Rihab Ameri, José Luis García, Amel Bouanane Derenfed, Nathalie Pradel, Sawssan Neifar, Sonia Mhiri, Monia Mezghanni, Nadia Zaraî Jaouadi, Jorge Barriuso, Samir Bejar

**Affiliations:** 1 Laboratory of Microbial Biotechnology, Enzymatic and Biomolecules, Centre of Biotechnology of Sfax (CBS), University of Sfax, Sidi Mansour Road Km 6, P.O. Box 1177, 3018 Sfax, Tunisia; 2 Department of Microbial and Plant Biotechnology, Centro de Investigaciones Biológicas Margarita Salas, Consejo Superior de Investigaciones Científicas (CIB-CSIC), C/ Ramiro de Maeztu 9, 28040 Madrid, Spain; 3 IBISBA_ES_CSIC_Cell Factory_MM, Madrid, Spain; 4 Laboratoire de Biologie Cellulaire et Moléculaire (Équipe de Microbiologie), Université des Sciences et de la Technologie Houari Boumédiènne, Bab Ezzouar, Algiers, Algeria; 5 Université de Toulon, CNRS, IRD, MIO, Aix Marseille Univ, Marseille, France

**Keywords:** *Caldicoprobacter algeriensis* TH7C1^T^, CAZymes, CGC, Biotechnological applications

## Abstract

**Background:**

Omics approaches are widely applied in the field of biology for the discovery of potential CAZymes including whole genome sequencing. The aim of this study was to identify protein encoding genes including CAZymes in order to understand glycans-degrading machinery in the thermophilic *Caldicoprobacter algeriensis* TH7C1^T^ strain.

**Results:**

*Caldicoprobacter algeriensis* TH7C1^T^ is a thermophilic anaerobic bacterium belonging to the Firmicutes phylum, which grows between the temperatures of 55 °C and 75 °C. Next generation sequencing using Illumina technology was performed on the *C. algeriensis* strain resulting in 45 contigs with an average GC content of 44.9% and a total length of 2,535,023 bp. Genome annotation reveals 2425 protein-coding genes with 97 ORFs coding CAZymes. Many glycoside hydrolases, carbohydrate esterases and glycosyltransferases genes were found linked to genes encoding oligosaccharide transporters and transcriptional regulators; suggesting that CAZyme encoding genes are organized in clusters involved in polysaccharides degradation and transport. In depth analysis of CAZomes content in *C. algeriensis* genome unveiled 33 CAZyme gene clusters uncovering new enzyme combinations targeting specific substrates.

**Conclusions:**

This study is the first targeting CAZymes repertoire of *C. algeriensis*, it provides insight to the high potential of identified enzymes for plant biomass degradation and their biotechnological applications.

**Supplementary Information:**

The online version contains supplementary material available at 10.1186/s12934-022-01818-0.

## Background

The Carbohydrate Active Enzymes (CAZymes) are enzymes involved in the assembly, modification or deconstruction of carbohydrates [1]. Based on amino acid sequence similarities, CAZymes are divided into several classes, including glycosyltransferases (GT) [2, 3], glycoside hydrolases (GH) [4–6], polysaccharide lyases (PL) [7, 8], carbohydrate esterases (CE) [8], and auxiliary activities (AA) [9] that have been stored in the CAZy database. The huge diversity of natural glycans and their complexity has boosted studies uncovering novel CAZymes. Thus, the number of CAZymes families increases exponentially by about four new GH families per year [10]. This broad diversity has allowed their use in plenty of industrial applications as they have been described to offer attractive opportunities in a wide range of biotechnological applications such as animal feed, biocatalysis, agriculture, biorefinery, glycoengineering and biobleaching industries [11–16]. Along with classical methods, various omics approaches are presently applied in the field of biology for the discovery of potential CAZymes. This “omics” technologies include proteomics, transcriptomics, metagenomics, metabolomics and whole genome sequencing [13, 17, 18].

The systematic genome sequencing has largely fueled the discovery of novel plant biomass degrading enzymes [10]. Studies have shown that bacteria and fungi are the main producers of CAZymes in nature. Among them extremophilic microorganisms have received special attention because of their capacity to live in extreme conditions such as high temperature, pressure, alkalinity, acidity, or salinity, thanks to their corresponding extremozymes [19]. Owing to their robustness, extremozymes are capable to function under harsh conditions more effectively than enzymes from other microorganisms [20]. Accordingly, thermophilic enzymes offer great potential for application in biotechnology, opening the possibility of performing biocatalysis at higher temperatures that can be more beneficial in some industrial settings [21, 22]. Thus, the study of thermophilic microorganisms have emerged during recent years including genome profiling and exploration of CAZymes content [23]. It has been demonstrated that carbohydrate acting enzymes works in conjunction with other CAZymes and proteins forming clusters of physically linked genes called polysaccharide utilization loci (PULs) [24–26]. These clusters that occur in bacteria of bacteroidetes phylum have been progressively identified in firmicutes phylum [27].

The thermophilic anaerobic *Caldicoprobacter algeriensis* TH7C1^T^ strain was isolated from the hydrothermal hot spring of Guelma. It was classified as a novel species in *Calidicoprobacter* genus [28] and was demonstrated to produce some thermophilic enzymes [29, 30]. However, its exploitation, in particularly discovery of enzymes content such as CAZymes, was hampered by culture limitations, anaerobic and high optimal temperature (65 °C).

In order to understand plant biomass-degrading machinery and to discover new potential interesting CAZymes for biotechnological applications, we report, for the first time, the genome sequence of *C. algeriensis* TH7C1^T^*.* Furthermore, we report the prediction of CAZyme encoding genes as well as the identification of clusters acting on polysaccharides.

## Results

### Genome sequence and analysis

**Fig. 1:**
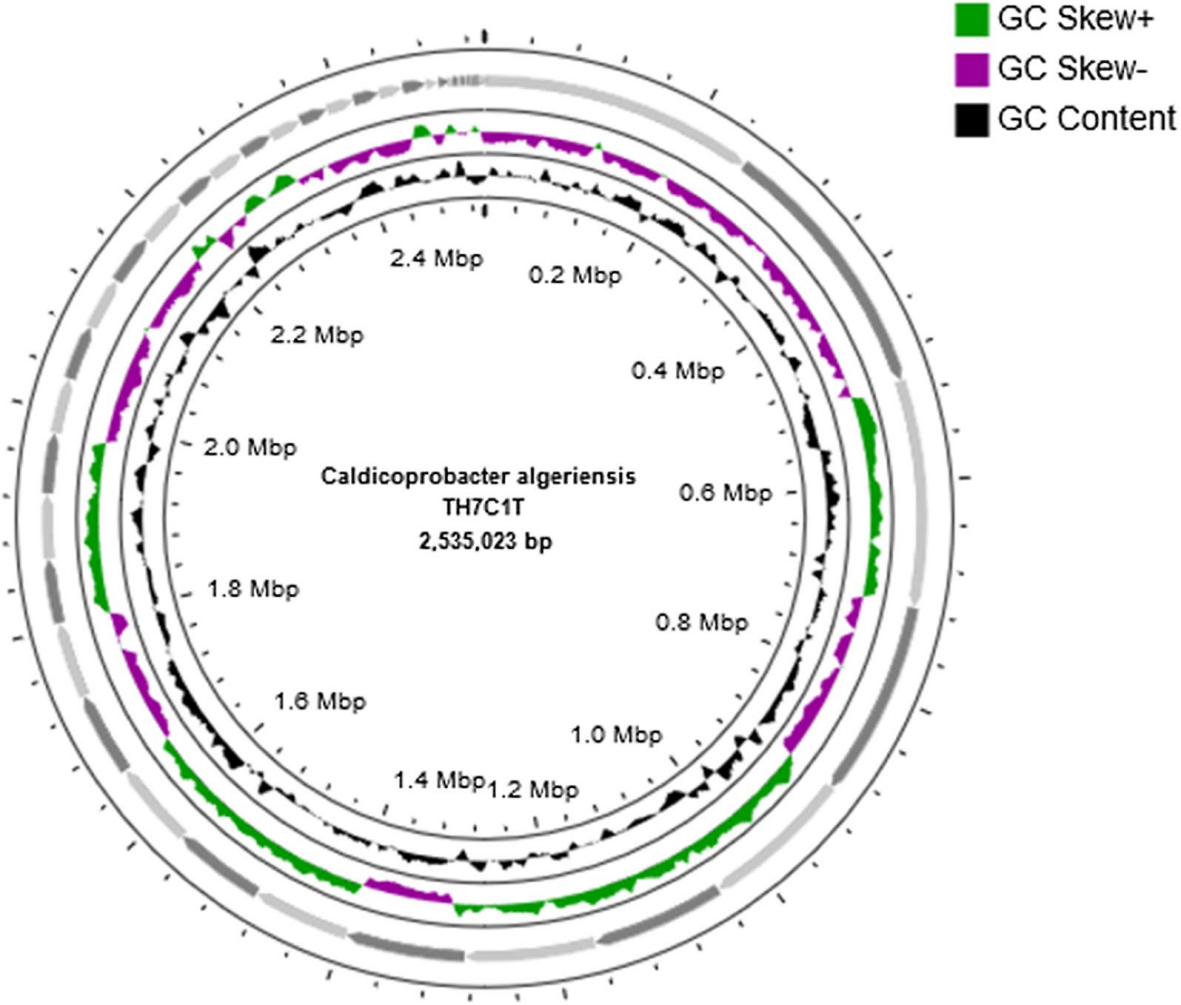
Graphical Circular genome map of *Caldicoprobacter algeriensis* TH7C1^T^ generated using CGView. From outside to inside, ring 1 represents the 45 assembled contigs. Ring 2 shows the GC skew with green indicating positive values and purple indicating negative values. The GC content is represented by the inner most ring

The genome sequencing of *C. algeriensis* TH7C1^T^ rendered 473,434 Illumina reads with an average coverage of 34.55x. The de novo assembly resulted in 45 contigs and a total length of 2,535,023 bp (Accession number PRJNA743054) with an overall GC content of 44.9%. A circular genome map of *C. algeriensis* was constructed, showing contigs, GC content, and GC skew (Fig. 1).

The overall genome statistics of *C. algeriensis* are close to those from *Caldicoprobacter faecalis, Caldicoprobacter oshimai* and *Caldicoprobacter guelmensis* (Table 1).

**Table 1:** Comparison of genome features between *C. algeriensis, C. faecalis, C. oshimai* and *C. guelmensis*

*Caldicoprobacter* species	*C. algeriensis*	*C. faecalis*	*C. oshimai*	*C. guelmensis*
Total Sequence Length (bp)	2,535,023	2,579,145	2,693,766	2,398,524
Number of contigs	45	87	427	20
Longest Sequences (bp)	255,969	158,877	–	505,220
N50 (bp)	121,172	78,525	10,681	394,366
GC content (%)	44.9	44.9	45.4	43.7
Number of CDSs	2425	2337	2618	2150
Average Protein Length	297.1	–	–	–
Number of RNAs	53	62	71	60

**Fig. 2:**
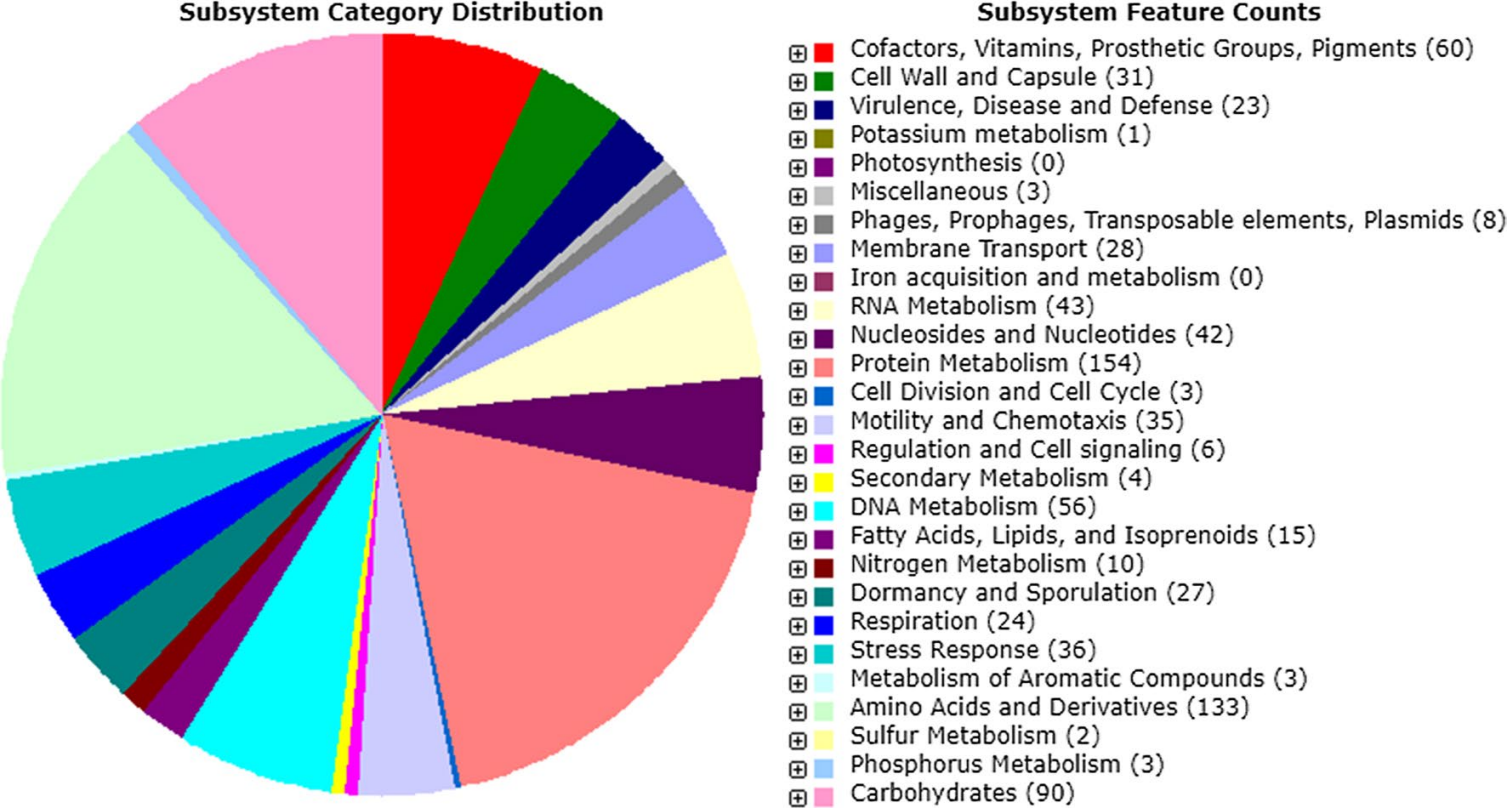
An overview of the RAST annotation and subsystems distribution for the *C. algeriensis* genome

Gene prediction performed with the RAST server resulted in 2720 features including 2666 protein coding sequences (CDSs) classified in 226 SEED subsystems and 53 RNA genes. Figure 2 shows the subsystem category distribution following RAST annotation. The largest part of this subsystem is allocated to the Amino Acids and Derivatives, and Carbohydrate metabolism with 15.83% and 10.71%, respectively. Dfast annotation revealed 2425 protein coding sequences with CDSs and 53 RNA genes covering 85.3% of the genome, the average length of the CDSs is 297 bp.

Analysis of genome stability using RAST and the CRISPRCasFinder server revealed two CRISPR array sequences located in contig 13 (with the evidence level of 4) and contig 2 (1 evidence level). This analysis revealed also three Cas cluster sequences detected in contig13 and related to CAS, CAS-TypeIIID and CAS-TypeIB. Another Cas cluster belonging to CAS-Type IE is located in contig 12.

### CAZymes annotation

Sequences submitted to the dbcan server allowed the automated annotation of CAZymes using the HMMER3.0 package and the dbcan CAZyme database (see Additional file 1: Table S1). This analysis resulted in 97 genes associated with glycan assembly and breakdown. The most abundant enzymatic family predicted in this genome was glycoside hydrolases with 57 CAZyme encoding genes divided into 32 different families.

The highest number of glycoside hydrolases found in *C. algeriensis* was related to GH109 with 9 predicted encoding genes, followed by GH3 with 6 genes and GH2/GH13 with 4 genes. GH109 family, which contains members involved in the deconstruction of galactomannans was widely represented in this genome. Interestingly, CAZymes belonging to this family had not been identified as major catalysts in previous studies highlighting biomass-degrading potential in hot spring. The GH3 family is represented by 6 predicted enzymes for hemicellulose hydrolyzing and debranching activities such as glucosidase, xylosidase and glucanase. Interestingly, GH3 has been reported as the most abundant GH family for oligosaccharides degradation in hot spring ecosystems [31]. The other abundant glycoside hydrolases predicted in this genome were identified to belong to the GH2 and GH13 families catalyzing the degradation of oligosaccharides and starch, respectively.

The second most frequent enzyme family contained in this genome is the glycosyltransferases GT family (20 encoding genes). GTs are known to catalyze the transfer of sugar residues from activated donor molecules to saccharide or non-saccharide acceptor molecules to form glycosidic linkages. The finding corroborates the results of biomass-degrading enzyme potential exploration in hot spring ecosystems previously reported [31] demonstrating that glycoside hydrolases and glycosyltransferases are widespread groups of CAZymes present in thermophilic microbial communities.

The output from dbCAN2 also included multiple hits corresponding to carbohydrate esterases (CEs) represented with 6 predicted genes attributed to CE1, CE4 and CE9 families. CEs are enzymes acting on ester bonds in carbohydrates accelerating the degradation of polysaccharides and facilitating the access of glycoside hydrolases. The most abundant CEs in *C. algeriensis* genome belong to CE4 family acting on acetylated xylan and chitin. Members of CE1 and CE9 families are involved in xylan and acetylglucosamine hydrolyzing, respectively.

The remaining putative CAZyme detected has been attributed to polysaccharide lyases (PL) represented by only one predicted gene. This genome also encodes 14 carbohydrate-binding modules (CBM). The majority of predicted CBMs belong to CBM4 and CBM50. CBM4 encodes specific modules that recognize xylan, 1,3-glucan, 1,3-1,4-glucan, 1,6-glucan, and amorphous cellulose, while CBM50 proteins are responsible for binding of enzymes having cleavage activity of chitin or peptidoglycan. They were found associated to GH genes or other CBMs. CAZyme genes prediction as well as the protein encoding genes sequences are available in supplementary (Additional files 1: Table S1 and 2).

Fast blast hit of CAZyme encoding genes in the CAZy database was performed by querying the genome against DIAMOND from dbcan meta-server. This analysis showed an identity between 35 and 83% with their nearest neighbors (Table 2).

**Table 2:** Comparison of predicted CAZymes of *C. algeriensis* with those available in CAZy database using DIAMOND tool in dbCAN

Query ID	Best Blast hit ID	% identical	Length	Query Start	Query End	Subject Start	Subject End	E Value
NODE_1_98	AFM44649.1|GH3|3.2.1.37|3.2.1.37	69.6	782	4	782	9	788	0.0e+00
NODE_1_142	QBQ55251.1|CBM50|	41.6	878	1	867	19	889	4.3e−173
NODE_2_26	CAN65674.1|GH28|	51.4	552	73	618	111	642	2.2e−158
NODE_2_32	AWP04987.1|GT13|	37.9	939	10	854	100	1037	6.2e−172
NODE_2_34	AUS96821.1|GH4|	83.7	436	3	438	2	437	4.1e−225
NODE_2 _43	QAV32893.1|GH3|	65.8	719	6	717	4	722	4.0e−278
NODE_2_48	QTM98178.1|GH35|	40.1	770	8	770	2	757	1.1e−172
NODE_2_73	QEX52072.1|GH29|	70.4	422	1	422	3	424	2.3e−196
NODE_2_91	QBQ55156.1|CBM50|	36.6	658	164	820	62	707	2.3e−112
NODE_2_93	ADH60031.1|GH67|	76.9	684	5	686	10	690	0.0e+00
NODE_2_112	ADH61394.1|GH127|	58.5	650	5	651	8	648	1.7e−235
NODE_2_119	AZR73767.1|GH1|	63.7	443	6	447	6	445	2.3e−178
NODE_2_195	QTH41126.1|GH30_1|	62.0	447	1	445	2	447	1.0e−175
NODE_2_205	QGG57161.1|GH2|	63.2	560	1	560	1	550	4.9e−221
NODE_3_3	AQQ10327.1|CBM38|GH32|	47.2	557	11	538	201	751	9.7e−152
NODE_3_5	BBH23071.1|GH151|	37.5	677	4	672	2	670	1.1e−147
NODE_3_18	AEE96970.1|GH109|	78.1	375	3	377	2	374	1.2e−180
NODE_3_20	AEE96984.1|GH148|GH30|	62.0	900	28	922	1290	2181	0.0e+00
NODE_3_28	QHT63269.1|GH28|	44.3	422	19	438	31	450	1.1e−103
NODE_3_33	AEE96972.1|GH106|	53.6	1009	1	1007	1	999	0.0e+00
NODE_3_34	ALS29147.1|GH140|	76.0	437	3	439	8	442	1.3e−213
NODE_3_ 39	AEM77894.1|GH2|	60.4	835	1	834	1	823	0.0e+00
NODE_3_88	AHO16406.1|GH13_11|	61.0	367	9	371	3	367	7.4e−123
NODE_3_137	SNX53392.1|GH42|	72.2	684	5	688	3	686	0.0e+00
NODE_3_151	AEE96723.1|GH3|	56.8	368	119	486	101	468	1.1e−120
NODE_4_38	SMX54901.1|GH38|	59.6	1026	1	1024	1	1022	0.0e+00
NODE_4_77	CCO21038.1|GH11|3.2.1.8| 3.2.1.8	52.3	622	5	625	24	634	3.7e−195
NODE_4_111	QNB44994.1|CBM34|GH13_39|	60.7	646	6	650	5	649	2.6e−247
NODE_4_112	QCX33357.1|GT5|	63.8	475	2	476	1	472	6.2e−182
NODE_4_113	QCX33360.1|CBM48|GH13_9|	70.6	622	8	629	4	620	2.1e−278
NODE_5_24	AXM88146.1|GH65|	52.0	782	1	777	1	778	1.1e−241
NODE_5_25	QUH28797.1|GH13_18|	56.8	555	18	572	8	553	7.1e−185
NODE_5_27	AUS95782.1|GH51|	73.7	490	1	490	1	490	4.9e−235
NODE_5_125	AZN38808.1|PL33_1|	48.1	626	1	626	1	623	4.6e−185
NODE_6_132	QAT62855.1|GT8|	64.6	268	1	268	1	268	2.6e−103
NODE_7_1	AEF18092.1|GH106|	65.9	1005	1	1001	1	1001	0.0e+00
NODE_7_17	AWZ17133.1|GH13_30|	41.4	1205	4	1159	406	1579	1.2e−247
NODE_8_2	AJO67863.1|GH3|	71.2	712	20	731	11	721	0.0e+00
NODE_8_11	AEE96371.1|GT51|	43.5	839	6	830	13	822	1.7e−183
NODE_8_27	ABB14581.1|GT51|	40.4	774	38	801	11	757	2.1e−153
NODE_8_48	QCW79542.1|GH101|	54.6	504	12	515	583	1068	1.4e−155
NODE_9_39	QSL64261.1|GT58|	39.1	504	15	480	202	683	4.7e−105
NODE_9_41	AEE95239.1|GH38|	61.6	921	19	938	42	961	0.0e+00
NODE_9_43	AEE97446.1|GH151|	53.7	659	4	657	6	662	1.5e−215
NODE_9_45	QGT51146.1|GT28|	55.1	365	1	364	1	365	3.1e−117
NODE_9_60	ACX65278.1|GH33|	56.9	334	1	328	1	323	1.5e−110
NODE_10_1	ADU28965.1|CBM4|GH16_3|	43.2	844	5	729	500	1333	8.0e−177
NODE_10_ 30	QVK18514.1|GH0|	30.4	828	50	855	61	856	3.9e−110
NODE_10_34	AXR85444.1|GH9|3.2.1.151|3.2.1.4|3.2.1.6|3.2.1.151|3.2.1.4|3.2.1.6	54.9	539	153	685	1	533	4.1e−171
NODE_10_35	AEE95681.1|GH10|	80.6	340	35	374	39	378	1.5e−175
NODE_10_41	QSQ07709.1|GH94|GT84|	60.2	2866	1	2865	1	2857	0.0e+00
NODE_11_1	AEE95836.1|GH116|	73.2	880	1	879	1	876	0.0e+00
NODE_14_60	AEV67980.1|GT4|	70.3	391	2	392	1	391	1.4e−163
NODE_17_1	QCT01556.1|CBM4|CBM54| GH16_3|	71.5	256	199	454	440	695	1.1e−111
NODE_17_20	AXG41146.1|GH6|	35.3	842	6	828	12	840	5.5e−128
NODE_17_35	AFM44649.1|GH3|3.2.1.37 |3.2.1.37	68.6	784	4	784	9	789	0.0e+00
NODE_18_30	AXG41146.1|GH6|	38.4	852	6	782	12	848	1.3e−128
NODE_18_36	BBH86227.1|GH2|	60.4	1038	10	1041	11	1025	0.0e+00
NODE_19_14	AEE95407.1|GH2|	73.7	582	1	582	1	571	2.1e−269
NODE_21_5	SNV81985.1|GT28|	56.5	352	2	353	3	353	1.3e−117
NODE_22_2	BAZ22920.1|GT4|	60.8	357	1	357	451	807	1.3e−131
NODE_23_13	AUS96695.1|GH31|	78.6	774	1	774	1	773	0.0e+00
NODE_25_1	QUI22575.1|CBM50|	38.5	589	7	593	34	621	9.5e−124
NODE_25_5	QMV43391.1|GH95|	54.6	799	13	804	8	787	1.2e−259
NODE_26_23	AEE96522.1|GH50|	51.8	467	1	464	65	528	4.2e−143
NODE_30_9	AEM77732.1|GH3|	74.1	509	1	508	17	525	3.5e−220
NODE_31_9	VUW72999.1|AA1|	51.0	451	166	612	182	620	8.4e−115

### PUL annotation and CGC prediction

To examine the presence of Gram-positive polysaccharide utilization loci (gpPUL) in the genome of *C. algeriensis*, we used nucleotide Basic Local Alignment search tool (BLASTX) available in dbCAN-PUL. This tool uses the repository as a database to query sequences against PUL proteins in dbCAN-PUL. This analysis resulted in a huge number of sequence similarities (11,320) (see Additional file 1: Table S2) including 36 CAZymes, 21 transporters (TCs) and 6 signal transduction proteins (STP). The PUL showing the highest number of hits to our query sequences is PUL0390 with a total of 10 hits. This PUL is predicted to be capable of degrading acetylated glucuronoxylan.

**Fig. 3:**
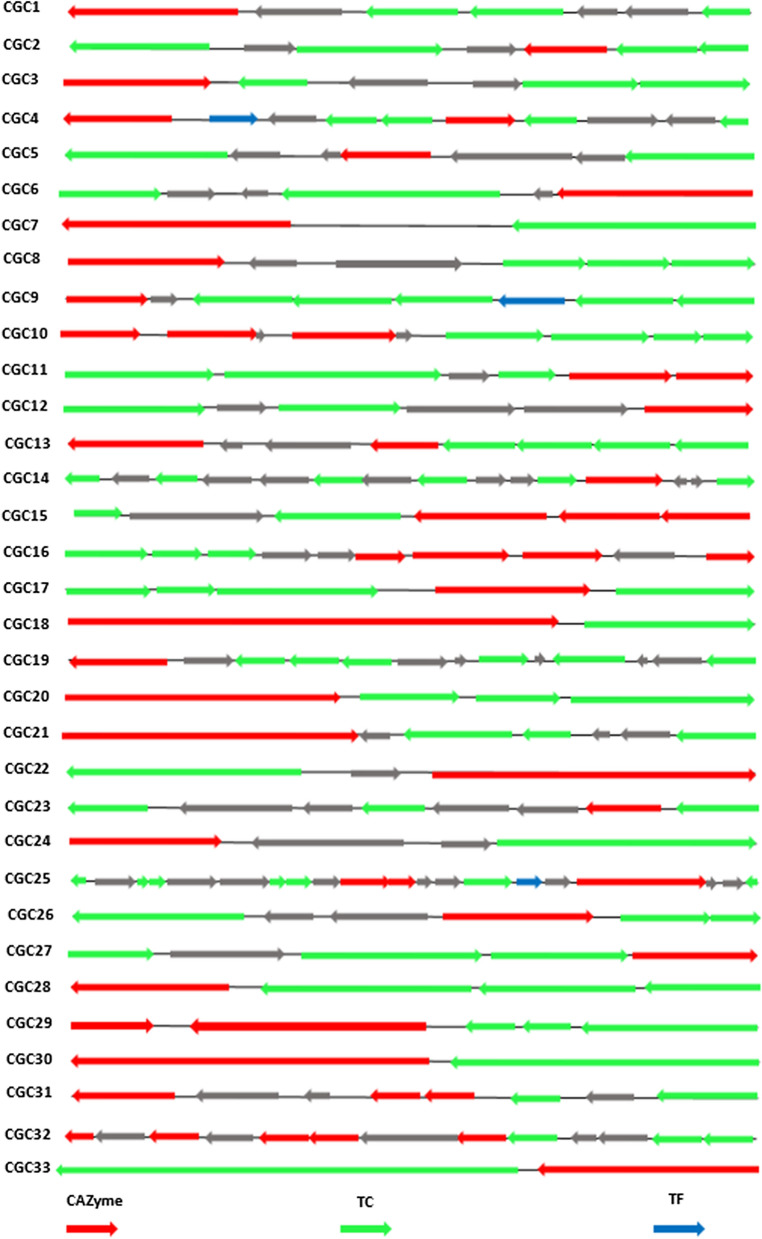
Schematic representation of the predicted 33 CAZyme Gene Clusters (CGCs) showing organization of genes in each cluster. CAZymes genes are colored red, TC (Transporters Classification) are colored green, TF (Transcription Factor) are colored blue. Non-signature genes, which can be inserted between signature genes, are colored gray

CAZyme gene clusters (CGC) prediction via the dbCAN2 with the CGC-Finder unveiled 33 CGCs defined by the presence of at least one CAZyme, one transporter and one transcription factor encoding genes (Fig. 3 and Additional file 1: Table S3).

CAZymes gene labels are based on CAZyme domain assignment, TC genes were predicted by searching against the TCDB and TF genes searched against the transcription factor families in Pfam and Superfamily. Genes organization of predicted clusters is shown in Fig. 3.

Results of sequence similarities were used for the determination of carbohydrate utilization ecotypes. Among the predicted CGCs, 20 of them contain CAZymes with no similarity with proteins in the repository. Based on enzymes combination in predicted CGCs and genes similarities with those available in dbCAN-PUL database, we predict a specific polysaccharide for each cluster (Table 3). The determination of carbohydrate utilization ecotypes provides insight to their biotechnological potential.

**Table 3:** Targeted substrates predicted for CAZymes genes clusters

CGC	Predicted CAZyme family	Targeted substrates
1	GH3	Xylan
2	CE19	Pectin
3	GH4	Melibiose, raffinose
4	GH3-GH35	Xylan, galactan
5	CE1	Xylan
6	GH67	Arabinoxylan
7	CBM25	Starch
8	GH30-1	Beta glucan, xylan
9	GH2	Hemicellulose
10	CBM66-GH32-GH151	Fructans
11	GH109-GH109	–
12	GH3	Chitin, xylan
13	GH109-GH109	–
14	GH38	Alpha mannan
15	GH13_39/CBM34-GT5-GH13_9/CBM48	Starch, glycogen
16	GH109-GH65-GH13_18-GH51	Arabinan
17	PL33-1	*N*-glycan
18	GH106	Rhamnose
19	AA1	–
20	GH3	Xyloglucan
21	GT51	–
22	GT51	–
23	GT2	–
24	GH109	–
25	GH5-25/GH9-GH10-GT84/GH94	Cellulose, hemicellulose
26	GH109	–
27	CBM50	–
28	GH109	–
29	GH109-GH2	Hemicellulose
30	GH2	Hemicellulose
31	GT4	–
32	GT2-GT4-GT4-GT2	Polysaccharides, *O*-antigen
33	GH23	peptidoglycan

## Discussion

Extremophilic microorganisms are of prime interest for biotechnological applications. They possess great potential to degrade plant biomass thanks to their corresponding enzymes [20]. Previous studies have shown that they are efficient producers of CAZymes [32, 33]. In the present work, we gained insight into the profile of genes involved in the carbohydrate metabolism (CAZomes) in the thermophilic and anaerobic *Caldicoprobacter algeriensis* TH7C1^T^. This strain classified as novel species in the *Caldicoprobacter* genera, was isolated from a hot spring. Owing to its harsh culture conditions, we proceeded with the whole genome sequencing to unveil the capability of *C. algeriensis* strain for polysaccharides utilization using complex machineries including efficient carbohydrate active enzymes. The *C. algeriensis* TH7C1^T^ genome consists of 2,535,023 bp with 44.9% GC content, which is similar to already sequenced *Caldicoprobacter* species, namely *faecalis*, *oshimai* and *guelmensis*.

In this study, we report for the first time, CAZymes repertoire of a thermophilic bacteria assigned to the *Caldicoprobacter* genera. The CAZymes prediction via the dbCAN server using predicted amino acid sequences of *C. algeriensis* unveiled the presence of 97 CDSs belonging to CAZymes representing 4% of protein coding genes. This percentage is within the range of CAZymes encoding-genes estimated for all microorganisms genomes [1] and genomes of previously reported thermophilic Firmicutes, such as BZ3 isolated from a new thermophilic compost-derived consortium (4%) [34], the thermophilic bacterium *Caldanaerobacter* sp. strain 1523vc isolated from a hot spring of Uzon Caldera (3,6%) [35]. Among predicted CAZymes, the most abundant class was glycosides hydrolases (GH), about 58% of CAZymes showing the highest percentage of Glycosidases reported in genomes and metagenomes from hot spring ecosystems. *C. algeriensis* also stands out for being the richest in diversity of GHs families (32) compared to other thermophilic genomes [34, 36, 37]. These GHs include the major families for hemicellulose and cellulose metabolism. Based on this, we speculate that *C. algeriensis* possess great potential to degrade carbohydrates much more effectively than other strains described previously.

When examining Glycosides hydrolase families by relative abundance, the maximum representation was from the families GH109 and GH3 genes. These two families are responsible for hemicelluloses and oligosaccharides biomass degrading respectively. As reported previously in thermophilic microbial consortia and hot spring samples, the other abundant class of CAZymes was glycosyl transferases (GT), 20% of predicted CAZymes. This large diversity of biomass degrading-related genes encoded by the *C. algeriensis* genome supports studies showing the importance of Firmicutes phylum in deconstruction of structural plant polysaccharides [27]. It has been demonstrated that this group of bacteria among the 6 predominant phyla in hot spring ecosystems [36, 38]. Given that they are nutritionally pecialized [27], they develop a battery of endo- and exo-acting Carbohydrate Active Enzymes and transporters, responsible for the cleavage of particular carbohydrates. Earlier studies reported that these genes are organized in clusters involved in polysaccharides degradation and transport forming Gram-positive polysaccharide Utilization Loci (gpPUL) [27]. In our study, we report for the first time the existing of CAZymes gene clusters in this group of Caldicoprobacteraceae.

PULs were analyzed based on genes homology with PULs available in dbCAN-PUL database. Results showed 11,320 gene similarities in CAZymes, transporters and signal transduction proteins across all PULs in the dbCAN repository, displaying an identity between 18.7% and 80.7%. To further analyze carbohydrate utilization ability of *C. algeriensis*, we performed CAZymes gene cluster analysis via the CGC finder in dbCAN2 meta server. We obtained 33 CAZymes gene clusters. Among them, 22 CGCs including 19 GH families, were predicted to be involved in cellulose and hemicellulose hydrolysis (GH3/GH5/GH2/GH10/GH30/GH35/GH38/GH4), glycogen degradation (debranching enzymes), (GH3/GH13_9/GH67/GH94) and starch utilization (GH13_39). The most abundant CAZyme identified in CGCs was related to the GH109 family. Nine genes, which typically encode α-*N*-acetylgalactosaminidase and β-N-acetylhexosaminidase, were found in seven clusters (CGC11, 13, 16, 24, 26, 28 and 29). GH109 genes were combined to other GH families genes, GH65/13/51 and GH2 in CGC 16 and 29 respectively, supporting that synergistic action of many CAZymes is required for polysaccharides cleavage [39]. Interestingly, analysis of GH109 genes similarities against genes from PULs available in the database, revealed no significant similarity. Thus, we suggest that *C. algeriensis* encodes new gene clusters not identified previously. Indeed, few studies reporting characterization of GH109 family members were performed [20] and CAZy database lists only 7 GH109 nagalases as characterized. Members of this family are particularly interesting for their ability to convert RBC A-antigens into H-antigens, turning type-A blood into universal donor type-O blood [40, 41].

The *C. algerinsis* also encodes six CAZymes genes clusters including members of GT families. As reported previously in extremophilic ecosystems, most of GT genes belonged to GT2 and GT4 families [20, 36, 42]. These two families have been reported to perform the synthesis of alpha and beta glycans and glycoconjugates. The GT4 contains a large variety of enzymes that are involved in lipopolysaccharide and antibiotic avilamycin A synthesis [43]. Owing the difficulty on purifying and investigating the biochemical features of these membrane associated enzymes, a few number of GTs has been characterized. Nevertheless, they have been described to offer potential opportunities in biotechnological applications such as biomedicine, cell biology field and pharmaceutical industry. Consequently, an in depth analysis of genes belonging to this family is very important.

Carbohydrates esterases are also identified in two CAZymes genes clusters (CGC2 and CGC5), related to CE1 and CE19 families. CE1 constitute the largest family of esterases including 5062 entries listed in CAZy database. Members of family CE1 were known to target xylan while CE19 family members are involved in pectin degradation. Recently, Carbohydrate esterases have shown great potential in several industrial applications such as food industry, pulp and paper industry, biofuel production, animal feed, medical and pharmacological industry [44, 45].

Genes Similarity analysis has shown 11 other genes, in addition to GH109, with no homologous in PULs database, including genes belonging to GT2, GT4 and CE19 CAZymes families. Thus, the *C.algeriensis* genome could be a source of novel and original thermophilic enzymes with strong potential for biotechnological applications.

## Conclusions

The present work constitutes the first study targeting CAZymes repertoire of bacteria belonging Caldicoprobacteraceae group based on whole genome sequencing. CAZyme encoding genes prediction results highlighted the high potential of *C.algeriensis* bacteria for the degradation of structural plant polysaccharides. Detailed analysis of predicted genes unveiled complex machineries involved in the metabolization of these major components of the plant cell wall and put the emphasis on newly identified enzymes. The in depth characterization of the specificity of each of these enzymes is the next challenge that will allow the understanding at the molecular level of the involvement of these loci in carbohydrates metabolism and their potential industrial applications.

## Methods

### Sampling and DNA extraction

Strain *C. algeriensis* TH7C1^T^ was isolated from the hydrothermal hot spring of Guelma [28]. Genomic DNA was extracted as previously described [46] with some modifications. Briefly, cells harvested in the exponential phase were suspended in TRIS–HCl (pH 8.0), EDTA, NaCl and incubated in the presence of lysozyme at 37 °C. Sodium dodecyl sulfate was added to 1% and the incubation continued until clarification was complete. Chloroform extractions were carried out and followed by ethanol precipitation. The DNA was drawn out of solution by being wound around a glass rod.

### Sequencing and functional annotation

The isolated DNA from *C. algeriensis* TH7C1^T^ was used to generate Illumina shotgun paired-end sequencing libraries, which were sequenced with a MiSeq instrument and the MiSeq reagent kit version 3 (2 × 250 bp paired-end reads), as recommended by the manufacturer (Illumina, San Diego, CA, USA) at IBISBA CSIC-CellFactory_MM platform. Quality filtering using Trimmomatic version 0.36 resulted in 473,434 paired-end reads rendering an approximate genome coverage of 30x. The sequence was assembled using the SPAdes Genome Assembler version 3.15.2. Assembled contigs were submitted to the Rapid Annotation Server (RAST) (http://www.nmpdr.org/FIG/wiki/view.cgi/FIG/RapidAnnotationServer) [47] and the DFAST server (https://dfast.nig.ac.jp/) for protein coding sequences (CDSs) prediction. The Circular Genome Viewer (CGView server) [48] was used to construct a circular graphical map of *C. algeriensis* TH7C1^T^. Carbohydrate-active enzyme (CAZyme) searches were performed using HMMER3.0 package (http://hmmer.org/) available from dbCAN (http://csbl.bmb.uga.edu/dbCAN/) [49], this search is run against Pfam Hidden Markov Models (HMMs). DIAMOND available from the dbcan CAZyme database was used for fast blast hits in the CAZy database.

Polysaccharides Utilization Loci (PULs) were analyzed via the dbcanPUL meta server [50]. CAZyme gene cluster (CGC) Finder in the database was used for carbohydrate-active enzyme clusters annotation. CGCs were defined as genomic regions containing at least one CAZyme gene, one transporter (TC) gene, and one transcription factor (TF) gene. Genome sequence has been submitted to the public genomic NCBI database under accession number PRJNA743054.

Prediction of CRISPR-Cas sequence (Clustered Regularly Interspaced Short Palindromic Repeats) in the genome was performed using the CRISPRCasFinder server) [51].

## Supplementary Information

**Additional file 1: Table S1.** Predicted CAZyme genes using the HMMER3.0 package and the dbcan CAZyme database. **Table S2.** Sequence similarities with PUL proteins in dbCAN-PUL. **Table S3**. Predicted CAZyme gene clusters (CGC) via the dbCAN2.

**Additional file 2.** Protein encoding genes sequences.

## Data Availability

All data generated or analysed during this study are included in this published article and its supplementary information files. The *C.algeriensis* Genome has been deposited in the public genomic NCBI database with accession code: PRJNA743054, (https://www.ncbi.nlm.nih.gov/bioproject/?term=PRJNA743054).

## Abbreviations

CAZyme: Carbohydrate Active Enzyme
GT: Glycosyltransferases
GH: Glycoside hydrolases
CE: Carbohydrate esterases
PL: Polysaccharide lyases
AA: Auxiliary activities
SPAdes: St. Petersburg genome assembler
RAST: Rapid Annotations using Subsystems
DFAST: DDBJ Fast Annotation and Submission Tool
CRISPR-Cas: Clustered Regularly Interspaced Short Palindromic Repeats
PUL: Polysaccharide Utilization Locus
gpPUL: Gram-positive Polysaccharide Utilization Locus
CGC: CAZyme Gene Cluster
CBM: Carbohydrate-binding module
TC: Transporter Classification
TCDB: Transporter Classification Database
STP: Signal Transduction Protein
TF: Transcription Factor
Pfam: Protein families database
HMMs: Hidden Markov Models
CDS: Coding sequence
dbCAN: DataBase for automated Carbohydrate-active enzyme ANnotation
